# Investigating the Role of Second Chance Schools and COVID-19 Pandemic on the Mental Health and Self-Image of Greek Adult Students

**DOI:** 10.3390/brainsci13081203

**Published:** 2023-08-14

**Authors:** Georgia Karakitsiou, Spyridon Plakias, Katerina Kedraka, Aikaterini Arvaniti, Christos Kokkotis, Anna Tsiakiri, Maria Samakouri

**Affiliations:** 1 Department of Psychiatry, Medical School, Democritus University of Thrace, 68100 Alexandroupolis, Greece; 2 Department of Physical Education and Sport Science, University of Thessaly, 38221 Trikala, Greece; 3 Department of Molecular Biology and Genetics, Democritus University of Thrace, 68100 Alexandroupolis, Greece; kkedraka@mbg.duth.gr; 4 Department of Psychiatry, University General Hospital of Alexandroupolis, 68100 Alexandroupolis, Greece; 5 Department of Physical Education and Sport Science, Democritus University of Thrace, 69100 Komotini, Greece; ckokkoti@affil.duth.gr; 6 Department of Neurology, Medical School, Democritus University of Thrace, 68100 Alexandroupolis, Greece; atsiakir@med.duth.gr

**Keywords:** mental health, self-image, generalized anxiety disorder, depression, well-being, self-esteem, self-efficacy, COVID-19, Second Chance Schools

## Abstract

COVID-19 has globally impacted both physical and mental health. This study aimed to explore the impact of Second Chance Schools (SCS) and the COVID-19 pandemic on the mental health and self-image of Greek SCS students. A total of 251 SCS students from two consecutive study cycles participated, completing the research instruments at the beginning and end of their studies. Participants’ anxiety, depressive symptomatology, well-being, self-esteem and self-efficacy were evaluated by means of the GAD-7, PHQ-8, WHO-5 Well-being Index, Rosenberg Self-Esteem Scale and Generalized Self-Efficacy Scale, respectively. The research spanned three years, including a year of universal lockdown, a year with protective measures and a year without anti-COVID-19 measures. Factor analysis, regression analyses and two two-way repeated measures ANOVAs were applied to the collected data. All five psychological dimensions measured by the study’s instruments were grouped into two factors, namely mental health and self-image. Well-being positively influenced mental health, while anxiety and depression had a negative impact. On the other hand, self-efficacy and self-esteem positively contributed to self-image. Mental health and self-image were moderately correlated. Pre-SCS values of mental health and self-image predicted a higher percentage of variance in post-SCS values compared to anxiety, depression, well-being, self-efficacy and self-esteem. Moreover, mental health improved after the completion of SCS, but only for participants after the lifting of anti-COVID-19 measures. Conversely, self-image improved for all participants regardless of the presence of anti-COVID-19 measures. Overall, the SCS had a considerable impact on the participants’ mental health and self-image, although the effect was influenced by COVID-19.

## 1. Introduction

In late 2019, a new coronavirus, called SARS-CoV-2, was identified as the cause of an outbreak of acute respiratory disease in the city of Wuhan, situated in Hubei Province, China. In February 2020, the World Health Organization (WHO) designated the disease as COVID-19 (Coronavirus Disease 2019) and declared it a public health emergency of international concern. In March 2020, COVID-19 was classified as a pandemic to emphasize its severity and urge countries to detect infections and prevent its spread [1,2]. Due to person-to-person transmission of the disease, quarantine, testing and a variety of social isolation measures were implemented globally, including in Greece [3,4,5,6,7,8,9]. On 5 May 2023, after over three years, the WHO Emergency Committee no longer considered COVID-19 a Public Health Emergency of International Concern [10].

Regarding COVID-19, similar to other pandemics, there were serious psychological effects on the population due to anti-COVID-19 measures, particularly related to quarantine and social isolation. Withdrawal and exclusion from others significantly affect individuals’ sense of well-being [11,12,13,14]. In particular, the anti-COVID-19 measures significantly impacted the global population’s mental health. Numerous global studies have identified elevated rates of mental difficulties, like depressive symptomatology, clinical anxiety, self-injury, suicidal ideation, sleep disturbance, cognitive problems, intrusive thoughts and ruminations, an increase in domestic violence and a worsening of quality of life [15,16,17,18,19,20,21,22]. The adverse impact of the pandemic on the psychological status of children, adolescents, university students and young adults in general has been specifically studied and demonstrated. Especially, exacerbation of existing mental health issues among children and adolescents has been attributed to the combined effects of the public health crisis, social isolation, school closures, limited outdoor activities and economic recession [23,24,25,26,27].

In addition to its impact on all other sectors, the COVID-19 pandemic has posed the greatest challenge and created the largest disruption of education systems in human history, affecting nearly 1.6 billion students across over 200 countries and disrupting traditional educational practices. School communities have experienced disproportionate impacts on psychological health and well-being due to disruptions in daily routines and social deprivation resulting from school closures [28,29]. Additionally, the lack of technological resources in both formal and non-formal education settings [30] and at home [31] means that many learners, particularly adults, faced additional barriers to completing their educational endeavors [32]. It seems that the pandemic has exacerbated pre-existing inequalities. Members of wealthier families as well as people already having digital expertise or access to specialized technological assistance had better outcomes in comparison to less privileged ones, in terms of their learning goals, well-being and mental health [33,34,35].

With regard to adult learners, Second Chance Schools (SCS) were established in Greece in 1997, after the recommendation of the European Commission for Education and Training. The SCS degree is equivalent to a basic education school diploma and is obtained within two academic years. The primary objective of SCS is to alleviate social and educational exclusion among disadvantaged and vulnerable groups by affording them a renewed opportunity to accomplish compulsory education. This is facilitated through innovative methods that enhance the acquisition of fundamental knowledge and the reinforcement of skills [36,37,38,39,40,41,42,43,44,45,46].

Regarding the profile of the students, it seems that they are characterized by low socioeconomic status and come from vulnerable, socially excluded and marginalized groups in almost all areas of public and private life [47,48,49]. Also, very often, these students face family problems, racial or cultural barriers, learning difficulties, possible mental illness and the lack of a supportive network [50,51,52]. In particular, in Greece, it seems that students face high levels of unemployment and are often living on the edge of poverty [53,54]. Students often have a negative self-image; low levels of self-esteem, self-efficacy and self-concept; and high levels of anxiety and depression, while experiencing negative emotions and constitute a “discouraged” group [55,56,57,58,59].

The current study is part of a wider research designed before the start of the pandemic and conducted in SCS in Greece during almost the entire time period of the COVID-19 pandemic. As far as we know, no longitudinal study has been published up to now that explores mental health or self-image in SCS or other student populations in Greece, covering the entire duration of the pandemic. Based on the literature, we hypothesized that attendance in SCS positively affects the mental health and self-image of the students, while the COVID-19 pandemic has a negative impact on these factors. This study aimed to explore the impact of Second Chance Schools (SCS) and the COVID-19 pandemic on the mental health and self-image of students. This study, along with others conducted during the COVID-19 pandemic, is deemed of significant importance, since, as can be seen from the literature, the psychological reactions of populations during pandemics play a crucial role in shaping the spread and occurrence of emotional and mental health problems during them [13,60].

## 2. Materials and Methods

### 2.1. Ethics

The study was approved by the following:

- The Ethics Committee of the Democritus University of Thrace (relevant document with protocol number 18,658/89 issued on 14 November 2019);
- The competent administrative body of the SCS in Greece, which is the Department of Adult Education Program and Organization Studies, which is under the Ministry of Education and Religious Affairs of the Hellenic Republic (relevant document with protocol number K1/20,484 issued on 12 February 2020).

### 2.2. Sample

The sample of this study consists of students from SCS based in the Region of Central Macedonia in Greece. From all the SCS of the specific region, through the method of simple random sampling [61,62], specifically using the lottery method, 7 school units were chosen to participate in the study. These were the SCS of Serres, the SCS of Naoussa, the SCS of Kordelio-Evosmos, the 2nd SCS of Thessaloniki, the SCS of Giannitsa, the SCS of Edessa and the SCS of Aridaia. It should be mentioned that the total number of school units that are located in the region of Central Macedonia is 19. The participation of students in the research was voluntary and anonymous. Before participating in the process, the students were given written informed consent to sign if they wished to participate. The criteria for taking part in the research were attending the specific school structures selected for the research and signing the written informed consent. The exclusion criterion was the refusal of the participants themselves. Furthermore, because in these specific school structures, there are immigrant students who do not speak the Greek language at all, they were excluded from the research.

### 2.3. Instruments

#### 2.3.1. Demographics

An ad hoc questionnaire was used to collect the following data: gender, age, family status, occupational status and number of children.

#### 2.3.2. Anxiety

The General Anxiety Disorder-7 (GAD-7) scale was used to detect anxiety, which is a tool for measuring generalized anxiety disorder and is completed by answering 7 items; the final assessment is indicated by the total score, which is calculated by summing up the scores of the 7-item scale. The possible answers range from 0 to 3, with the response categories of “not at all”, “several days”, “more than half the days” and “nearly every day”, respectively. The GAD-7 total score for the seven items ranges from 0 to 21 (0–4: minimal anxiety, 5–9: mild anxiety, 10–14: moderate anxiety, 15–21: severe anxiety) [63,64]. It is commonly used as a screening tool in primary care, and depending on the results, referral to a psychiatrist may be recommended [65]. Although designed as a screening tool for generalized anxiety, the GAD-7 also performs reasonably well as a screening tool for three other common anxiety disorders: panic disorder, social anxiety disorder and posttraumatic stress disorder [66].

#### 2.3.3. Depression

Regarding the assessment of depression, the Patient Health Questionnaire-8 (PHQ-8) scale was used; the 9th question concerning suicidality has been removed in this version. Scores of PHQ-8 and PHQ-9 are similar and the use of those tools yields similar research results [67]. The possible answers range from 0 to 3 to the response categories of “not at all”, “several days”, “more than half the days” and “nearly every day”, respectively. The PHQ-8 total score for the eight items ranges from 0 to 24 (0–4: no depression, 5–9: mild depression, 10–14: moderate depression, 15–20: moderately severe depression, 21–24 severe depression). This scale is a self-administered tool and is recommended for use in primary care and is widely utilized as a tool for the detection of depressive symptomatology in healthy populations [68,69]. It is worth noting that both the GAD-7 and the PHQ-8 were developed by Spitzer, Williams, Kroenke and colleagues. No license is required for their use as they are available online for free and translated into Greek by Pfizer Inc. [70].

#### 2.3.4. Well-Being

The WHO-5 Well-Being Index was used to measure well-being, which measures the individual’s current subjective sense of mental quality of life. The tool was created by the World Health Organization in 1998 and has been translated to and weighted in several languages, one of which is Greek. It consists of 5 questions that assess the quality of life in the current period and the subjective well-being of the respondents in the past 2 weeks. The scale has adequate validity both as a screening tool for depression and as an outcome measure in clinical trials and has been successfully applied in a wide range of study areas. The raw score is calculated by totaling the figures of the five answers. The raw score ranges from 0 to 25, with 0 representing the worst possible and 25 representing the best possible quality of life. To obtain a percentage score ranging between 0 and 100, the raw score is multiplied by 4. A percentage score of 0 represents the worst possible quality of life, whereas a score of 100 represents the best possible quality of life [71,72,73,74]. This questionnaire does not require permission to use [75].

#### 2.3.5. Self-Esteem

The Rosenberg Self-Esteem Scale (RSES) was used to measure self-esteem. It is a tool that calculates a person’s self-esteem. It consists of 10 questions, 5 of which are scoring positive and the other 5 are negative. The scale ranges from 0 to 30. Scores between 15 and 25 are within the normal range, while scores below 15 suggest low self-esteem. It is worth noting that the specific tool has been weighted in Greek and has demonstrated high reliability and validity [76], and its authentic version also has high validity and reliability [77]. This questionnaire does not require permission to use [78].

#### 2.3.6. Self-Efficacy

To investigate the students’ self-efficacy levels, the Generalized Self-Efficacy Scale was used [79], which includes 10 questions and statements and has been validated in the Greek population. These statements measure the individual’s subjective judgment of their general self-efficacy. To complete the process, the participants answered the questions on a scale from 1 to 4, where 1 indicates that something never happens and 4 indicates that it always happens. A total score can be calculated on a scale of 10 to 40. Higher scores indicate higher levels of perceived general self-efficacy, and lower scores indicate lower levels of perceived general self-efficacy. The specific tool has been translated to and weighted in the Greek language [80], and it has been shown to have high validity and reliability [81]. This questionnaire does not require permission to use [82].

### 2.4. Data Collection

The data were collected in two stages corresponding to two different study cycles. The first stage concerned the years 2020–2022 (Group 1) and the second stage concerned the years 2021–2023 (Group 2). Specifically, in the year 2020–2021, questionnaires were distributed in the A year of the first group, when the students started their studies at the SCS (A1 cycle, consisting of 132 people). In this particular year, education was provided remotely and asynchronously following a ministerial decision [83] in order to protect citizens from COVID-19. During the 2021–2022 school year, education was carried out closely with the particularity that unvaccinated students would have to carry out a rapid test for the detection of COVID-19 weekly in order to attend school. Furthermore, the entire student community and the teachers at the school had to wear medical masks throughout the lessons. This was ratified by ministerial decision number 4187/2021 [84]. In this particular year, questionnaires were distributed in the B year of the first study cycle (B1 cycle, consisting of 132 people), when the students were completing their studies at the SCS. Also, during this specific year, questionnaires were distributed in the A year of the second study cycle (A2 cycle, consisting of 119 people), when the students started their studies at the SCS. Finally, in the 2022–2023 school year, attendance at SCS, like all other school structures in the country, had no limitations regarding the handling of COVID-19 in accordance with ministerial decision number 2676/2022 [85]. During the specific school year, questionnaires were distributed in the B year of the second cycle of study (B2 cycle, consisting of 119 people), when the students were completing their studies at the SCS.

The questionnaires were completed anonymously by using a code of each student in order to correlate the questionnaires of the two years with each other. Questionnaires answered only in the first or the second year were not used because they did not meet the purposes of the research. All abbreviations and their definitions are presented in the Abbreviations (Table 1).

### 2.5. Statistical Analysis

PCA factor analysis was performed using 502 observations (251 questionnaires before and 251 after) on 5 quantitative variables (CAD7, WHO5, PHQ8, SELFEFF, SELFEST). Factor analysis is a dimensionality reduction method carried out by grouping variables that have a common meaning [86,87]. To determine the suitability of the data for the application of factor analysis, two tests were conducted [88]: (a) the Kaiser–Meyer–Olkin (KMO) test was used to assess the adequacy of the sample [89] and (b) Bartlett’s test of sphericity was used to determine whether the variables are sufficiently correlated in the correlation matrix [90]. A loading value of 0.70 was considered substantial for each factor selection [91], and Cattell’s Scree Test was used to determine the number of factors [92,93]. The eigenvalues of the factors confirmed the selection [94]. Both orthogonal (varimax) and oblique rotations were performed, and the component correlation matrix of the oblique rotation showed a moderate correlation between factors. Therefore, oblique rotation was used [89]. The resulting factor scores for the two factors (latent variables) were used for subsequent analyses.

Seven different simple linear regression analyses were then conducted to examine whether the values of the five original variables (CAD7, WHO5, PHQ8, SELFEFF, SELFEST) and the two latent variables (derived from the factor analysis) before the SCS can predict the values of the corresponding seven variables after the SCS [95,96].

Finally, two two-way repeated measures ANOVAs were performed to determine whether the SCS, the change in the COVID-19 measures and the interaction between them cause statistically significant changes in the values of the two latent variables [97,98]. Bonferroni correction was used for post hoc comparisons [97,99]. All analyses were performed using IBM SPSS statistical software (version 25.0), and the statistical significance level was set at *p* < 0.05.

## 3. Results

### 3.1. Descriptive Analysis of the Sample

The total number of participants who took part in the research was 251, of which 132 belonged to the number 1 cycle (A1, September 2020; B1, June 2022) and 119 to the number 2 cycle (A2, September 2021; B2, June 2023). From the total survey sample, 105 people were men (41.8%) and 146 were women (58.2%). Of these, most of them belonged to the age groups 36–45 (31.1%—78 people) and 46–55 (31.1%—78 people). All the other descriptive characteristics of the sample are shown in the Table 2.

### 3.2. Measuring the Reliability of Research Tools

The reliability of the research tools used was measured. Internal consistency reliability assesses the degree of homogeneity exhibited by a measurement instrument. This assessment is carried out through Cronbach’s α index. It is noted that values greater than 0.7 are considered satisfactory. All instruments were found to have high reliability in terms of internal consistency, with Cronbach’s α values between 0.791 and 0.886.

### 3.3. PCA Factor Analysis

**Figure 1:**
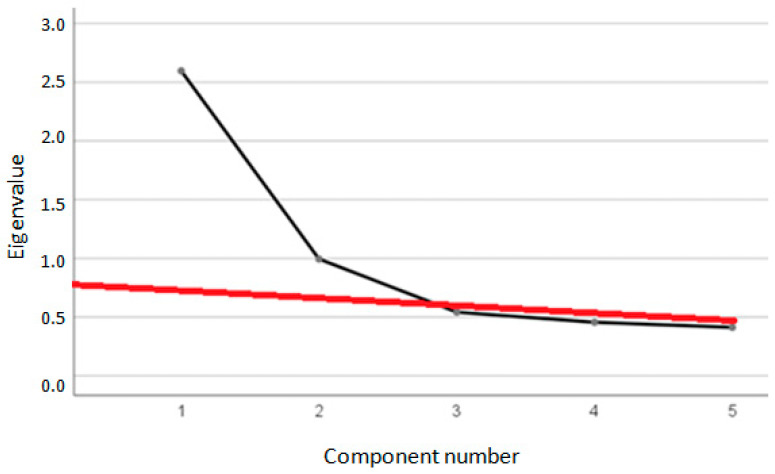
Scree plot. Black line is the standard scree plot, while red line is a Keelling’s regression line. This line approximates the cut-off eigenvalues. In our results, only the first two factors would be retained, since the others have eigenvalues below the regression line.

The Kaiser–Meyer–Olkin test and Bartlett’s test of sphericity indicated the suitability of the data for conducting factor analysis (KMO = 0.75; x^2^ = 666.63, *p* < 0.001). Figure 1 illustrates that two factors should be extracted.

Table 3 displays the eigenvalues of the factors, confirming that only the first two factors meet the Kaiser criterion. According to Turner (1998) and Ruscio and Roche (2012) [94,100], the difference between eigenvalues of 1.01 and 0.99 is negligible and may be attributed to sampling error. The same table reveals that the two factors account for 71.81% of the total variation (51.92% and 19.89%, respectively).

**Figure 2:**
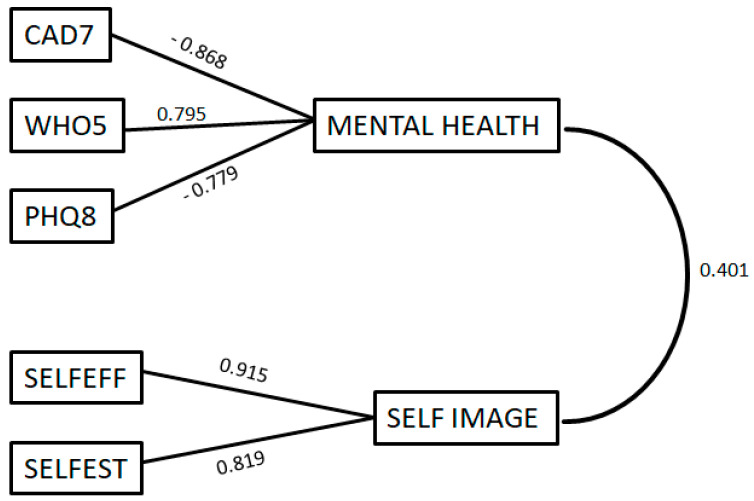
Representation of the loadings of the initial variables on the two extracted factors, as well as the correlation between the two factors.

Figure 2 presents the Pattern Matrix and the Component Correlation Matrix, displaying the loadings of the initial variables on the two factors as well as the correlation between the two factors.

### 3.4. Simple Linear Regression Analyses

Table 4 displays the variables used for the seven simple linear regression analyses, along with the corresponding unstandardized coefficients. These coefficients can be utilized to derive the prediction equation in the form of y = ax + constant, where “y” represents the value of the variable after SCS, “x” represents the value of the variable before SCS and “a” denotes the unstandardized coefficient B for the independent variable.

Table 5 shows that for the latent variables (compared to the five initial variables), a higher percentage of the variance in their post-SCS value is explained by their pre-SCS value. The largest proportion is observed in the SELF_IMAGE variable, where the pre-SCS value explains 81% of the variability in its post-SCS value.

### 3.5. Two-Way Repeated Measures ANOVA

**Figure 3:**
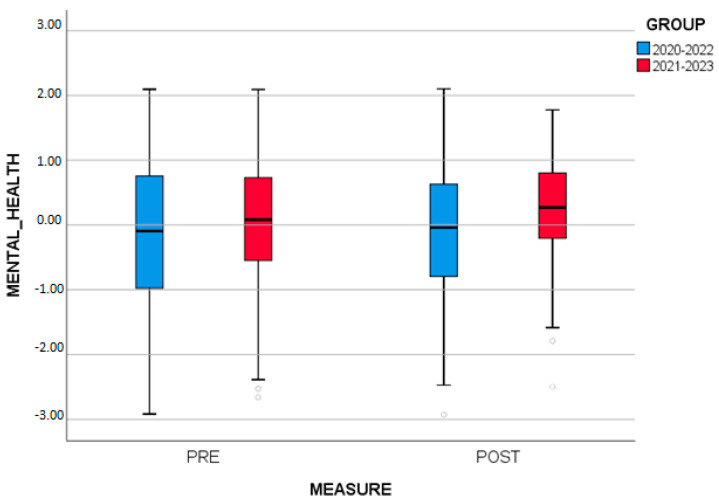
MENTAL_HEALTH by measure and by group.

Analysis of variance with one repeated factor and one independent factor (two-way repeated measures ANOVA) was applied to examine if there were differences in MENTAL_HEALTH between measurements (initial, final) and GROUPS (2020–2022, 2021–2023). The results show that there is a statistically significant interaction between measurements and GROUPS (*p* = 0.007). Analyzing the interaction in terms of measurement, it was found that there were no statistically significant differences in MENTAL_HEALTH between initial (pre) and final (post) in the group 2020–2022 (*p*= 0.883). On the contrary, statistically significant differences were found in MENTAL_HEALTH between initial (pre) and final (post) in the group 2021–2023 (*p* < 0.001). Examining the means, it appears that participants had a higher score in MENTAL_HEALTH after the SCS (M = 0.25 ± 0.81) compared to the initial measurement (M = 0.003 ± 1.01) (Figure 3).

**Figure 4:**
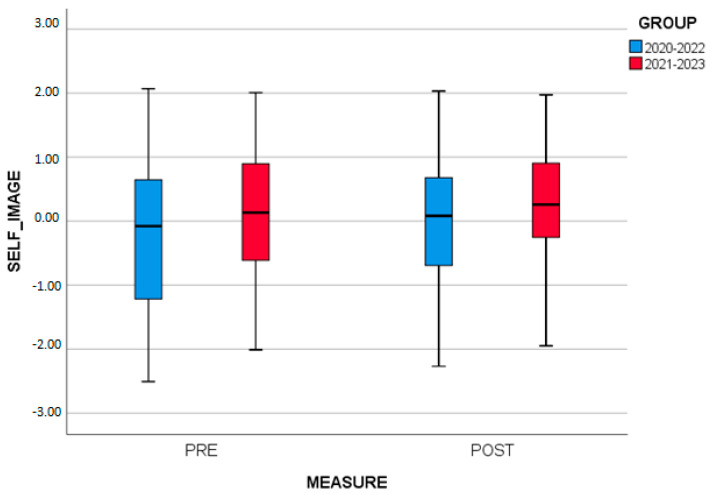
SELF_IMAGE by measure and by group.

Similarly, two-way repeated measures ANOVA was applied to examine whether there are differences in SELF_IMAGE between the measures (initial, final) and the GROUPS (2020–2022, 2021–2023). The results show that there is no statistically significant interaction between the measures and the GROUPS (*p* = 0.807). However, a significant main effect of the factor GROUPS (*p* = 0.036) was found, as well as statistically significant differences in the SELF_IMAGE between the initial and final measurement (*p* < 0.001). Examining the means, it appears that participants had a higher SELF_IMAGE score at the final measurement (M = 0.72 ± 0.95) than at the initial measurement (M = −0.72 ± 1.04) (Figure 4).

## 4. Discussion

The research was conducted in SCS in Greece, and a total of 251 students from two different study cycles participated. The participants were asked to fill out each questionnaire twice, once at the beginning of their studies and once at the end. The research spanned three years. In the year 2020–2021, Greece experienced a universal lockdown due to the pandemic. In the year 2021–2022, the pandemic continued, and courses in schools were conducted with anti-COVID-19 measures. However, in the year 2022–2023, there were no anti-COVID-19 measures in place. The aim of this study was to investigate the effect of the interaction between SCS and anti-COVID-19 measures on the anxiety, depression, well-being, self-efficacy and self-esteem of students in Greek SCS. The results of the PCA factor analysis revealed that the first three concepts are part of a broader concept called as mental health, while the last two concepts are related to self-image. Specifically, well-being positively contributed to mental health, while anxiety and depression had a negative impact. In contrast, both self-efficacy and self-esteem positively contributed to self-image. Furthermore, a moderate positive correlation was observed between mental health and self-image. Regression analysis demonstrated that the values of mental health and self-image prior to SCS predict a higher percentage of the variance in their values after SCS, compared to the corresponding percentage predicted by the values of anxiety, depression, well-being, self-efficacy and self-esteem. Lastly, the two-way repeated measures ANOVAs indicated that the mental health values of the participants after SCS differed from their values before the program, but only for the participants who completed school after the anti-COVID-19 measures were lifted. Conversely, participants’ self-image improved regardless of the presence of anti-COVID-19 measures.

The present research found that well-being, depression and anxiety are part of a broader construct called mental health. In particular, well-being positively contributes to mental health, while anxiety and depression have a negative impact. Similarly, in other research that was conducted, mental health resulted from the synthesis of the factors of anxiety, depression and well-being, such as the study by Bergersen et al., who investigated the mental health of patients 2 to 5 years after a stroke [101]. Another study examined the relationship between mental health and obesity by measuring the variables of anxiety, depression and emotional well-being [102]. Similarly, another study explored the effect of digital mental health interventions on college students, focusing on their anxiety, depression and psychological well-being [103]. In another instance, the relationship of anxiety, depression and well-being with physical activity was examined [104]. However, in other instances, mental health was found to be associated with anxiety and depression, but in combination with life satisfaction and positive affect [105]. In conclusion, as in the present research, the combination of anxiety, depression and well-being was used in other research to identify mental health. Especially anxiety and depression, either alone or with other factors, are frequently used as mental health indicators.

The present study found that self-esteem and self-efficacy are part of a broader construct called self-image. Specifically, both self-efficacy and self-esteem positively contribute to self-image. Similarly, in other research, self-esteem and self-efficacy were investigated in conjunction with the concept of self-personality to explore the entrepreneurial intention of individuals [106]. However, in other cases, these specific concepts (self-efficacy and self-esteem) were studied not in relation to self-image but as traits associated with narcissism [107]. In another case, they were associated with psychological well-being [108]. Furthermore, these two concepts were studied in combination with social support, body image and locus of control [109]. Finally, a study focused on self-efficacy alone, without considering self-esteem, to investigate self-image in adolescence [110]. In conclusion, as in the current research, the combination of self-efficacy and self-esteem has been used in other studies to identify self-personality traits.

Furthermore, the present study observed a moderate positive correlation between mental health and self-image. This means that adult learners with higher levels of mental health also had a better sense of self-image, and vice versa. This positive correlation has been observed in other instances. For example, a study investigated the effect of self-image and self-esteem on the mental health of African-American preteen girls and found a positive correlation between these factors [111]. Similarly, another study investigating these two factors in deaf and hard-of-hearing children attending either special schools or regular schools found a positive correlation between self-image and mental health [112]. Moreover, research has shown that higher levels of self-esteem had a buffering effect against the occurrence of mental health problems, especially depression and anxiety disorders, during the COVID-19 pandemic, as well as in non-pandemic conditions, confirming the interaction between the factors being studied [113,114,115]. This indicates that, as in our research, mental health and self-image interact with each other.

Regression analysis demonstrated that the values of mental health and self-image prior to SCS predict a higher percentage of the variance in their values after SCS, compared to the corresponding percentage predicted by the values of anxiety, depression, well-being, self-efficacy and self-esteem. No previous research has been found that was conducted in SCS and made such analyses between the above factors, making it an innovative aspect of the present study.

The present study employed two two-way repeated measures ANOVAs to examine the differences in mental health and self-image values of participants before and after SCS. The results showed significant differences only for the participants who completed the SCS after the anti-COVID-19 measures were lifted. Conversely, the present research showed that participants’ self-image improved regardless of the presence of anti-COVID-19 measures. Generally, many studies have reported students in SCS having high rates of possible mental health issues; negative self-image; low levels of self-esteem, self-efficacy and self-concept; and high levels of anxiety and depression, while experiencing negative emotions [50,51,52,55,56,57,58,59,116]. However, the above research only recorded the characteristics of the students, and it did not investigate whether the SCS influenced the development of the above variables. In one study, it was found that the graduates of SCS exhibited higher levels of self-esteem after completing their studies compared to individuals who have not completed their basic education. Furthermore, a positive correlation was found between self-confidence and self-image [117]. Another study showed that providing education to vulnerable populations leads to an improvement in their self-esteem [118]. Finally, a study explored the association between SCS, personality and psychological symptoms, examining whether SCS contributes to the well-being or psychological distress of the students [119]. Lastly, no literature has been found studying how attendance in SCS affects mental health, self-image, anxiety, depression, well-being, self-esteem or self-efficacy, making this another innovative aspect of the present research.

In our study, it was found that the anti-COVID-19 measures had a considerable impact on the participants’ mental health. Regarding the impact of COVID-19 on mental health, numerous studies found a significant correlation between the two factors, not only on individuals who contracted the virus but also on people who did not get sick but experienced the restrictive measures [120,121,122,123,124]. Research conducted among typical student populations found that the pandemic had a significant impact on stress, anxiety, depression and loneliness. Furthermore, it emerged that students from lower socio-economic strata and marginalized backgrounds experienced more severe consequences in their mental health, even during the early stages of the pandemic [104,125,126]. However, levels were found to return to normal around mid-2020 [127]. Similarly, numerous other studies have reported significant effects on students’ mental health due to the pandemic. More specifically, the studies found high rates of anxiety and depression [128,129], which in some cases were extremely high [130]. In the domain of adult education, specifically, it is evident that COVID-19 has worsened social inequalities and brought them into sharper focus, having implications for the well-being and mental health of these particular groups [32,131,132,133,134]. Moreover, regarding the factors of self-esteem and self-efficacy and the anti-COVID-19 measures, some studies did not find significant changes in the levels of those factors among the participants between anti-COVID-19 measures or afterward [135]. However, other studies have recorded lower levels of self-efficacy in underage students [136]. It should be noted that no research investigating the effect of the interaction between SCS and anti-COVID-19 measures on the mental health, self-image, anxiety, depression, well-being, self-esteem or self-efficacy of students was found, and this is another innovation of the present research.

The limitations of this study should be acknowledged. Firstly, the sample was derived from a single geographical region of Greece, specifically the region of Central Macedonia. Hence, it remains uncertain whether generalizable conclusions can be drawn for other populations. Secondly, during the years of conducting the research, a reduced number of participants in the specific school structures was observed, as reported by the school directors. Particularly, in the year 2021–2022, when unvaccinated students were required to undergo weekly rapid tests, lower enrollments were noted as many potential students declined to enroll due to financial constraints. This indicates that individuals belonging to genuinely vulnerable populations might not have been included in the sample.

## 5. Conclusions

Despite its limitations, the present research yielded significant insights into the effect of the interaction between the COVID-19 pandemic and SCS on individuals’ mental health and self-image. The study revealed that anxiety, depression and well-being are components of mental health, while self-efficacy and self-esteem are linked to self-image. Specifically, well-being positively impacts mental health, while anxiety and depression have a negative influence. On the other hand, self-efficacy and self-esteem positively contribute to self-image. Moreover, a moderate positive correlation was observed between mental health and self-image. The study also demonstrated that pre-SCS values of mental health and self-image predicted a higher percentage of their post-SCS values compared to anxiety, depression, well-being, self-efficacy and self-esteem.

The hypothesis was that attendance in SCS positively affects the mental health and self-image of the students, while the COVID-19 pandemic negatively affects these factors. The main findings of the present research indicated that participants’ mental health values after SCS differed from their values before the program, but only for those who completed the schools after the anti-COVID-19 measures were lifted. Conversely, participants’ self-image improved regardless of the presence of anti-COVID-19 measures. Overall, SCS alone improved both mental health and self-image, while anti-COVID-19 measures alone affected both aspects. In the SCS–anti-COVID-19 measures interaction, it was evident that SCS improved mental health only when anti-COVID-19 measures were not present, whereas it improved self-image regardless of the presence of such measures.

The study introduced several innovations. Firstly, we conducted a literature review but found no previous research predicting mental health and self-image values after SCS based on pre-SCS values. Additionally, no studies were found investigating the impact of SCS attendance on these factors, particularly in the context of the global COVID-19 outbreak. The research also stands out for its longitudinal approach, covering the entire duration of the pandemic instead of a single point in time. This enabled the examination of the evolving effects of the pandemic and SCS attendance on mental health and self-image among students.

The significance of this research, along with others of similar scope, lies in the crucial role that the psychological reactions of populations during pandemics play in shaping the spread and occurrence of emotional and mental health problems. Further studies could explore how these populations respond to other types of social changes and examine how SCS can support and strengthen vulnerable students in coping with social disturbances and challenges. Additionally, a holistic approach to mental health should be adopted, considering its close connection to prevailing societal conditions.

As it emerged, students in SCS come from vulnerable, socially excluded and marginalized groups, facing in many cases mental-health-related problems and issues regarding negative self-image. Education in the SCS contexts should aim to achieve reconnection with education and training systems and the acquisition of basic knowledge and skills. Furthermore, SCS, along with every school unit that provides basic education to adults, should invest more in preserving the mental health of students and improving their self-image. This can be achieved by implementing innovative processes that lead to a flexible curriculum and by focusing on the individual profiles of each adult student. Moreover, there is a need to utilize psychologists and employment consultants within SCS. These consultants should closely cooperate with the teachers association to detect possible problems emerging in their students and intervene using appropriate methods. Finally, SCS staff should always consider the prevailing social conditions when supporting the students.

## Figures and Tables

**Table 1:** Summary of the research stages, number of participants and measures to address the COVID-19 pandemic.

Academic Year of Distribution of Questionnaires	No. of Participants	Cycle	Conditions in Regard to COVID-19
September 2020–June 2021	132	A1	Universal lockdown
September 2021–June 2022	132	B1	Lessons with special
	119	A2	measures
September 2022–June 2023	119	B2	Non-COVID-19 year

**Table 2:** Participant demographics.

Demographics	Frequency	Percentage
Sex		
Male	105	41.8
Female	146	58.2
Total	251	100.00
Age group		
18–25	15	6
26–35	41	16.3
36–45	78	31.1
46–55	78	31.1
55+	39	15.5
Total	251	100.00
Marital status		
Married	131	52.2
Unmarried	56	22.3
Divorced	36	14.3
Widowed	11	4.4
In symbiosis	17	6.8
Total	251	100.00
Number of children		
0	74	29.5
1	37	14.7
2	85	33.9
3	37	14.7
4+	18	7.2
Total	251	100.00
Occupation		
Unemployed	111	44.2
Public employee	32	12.7
Working in the private sector	92	36.7
Retired	14	5.6
Other	2	0.8
Total	251	100.00

**Table 3:** Eigenvalues for components and total variance explained.

Component	Initial Eigenvalues			Extraction Sums of Squared Loadings			Rotation Sums of Squared Loadings
	Total	% of Variance	Cumulative %	Total	% of Variance	Cumulative %	Total
1	2.596	51.924	51.924	2.596	51.924	51.924	2.302
2	0.994	19.889	71.813	0.994	19.889	71.813	1.907
3	0.542	10.834	82.647				
4	0.455	9.108	91.755				
5	0.412	8.245	100				

**Table 4:** Dependent variable, independent variable and unstandardized coefficients B for each of the 7 simple linear regression analyses.

Number of Regression Analysis	Dependent Variable	Independent Variable	Unstandardized Coefficients B	
			Constant	Independent Variable
1	MENTAL_HEALTH_POST	MENTAL_HEALTH_PRE	0.091	0.622
2	SELF_IMAGE_POST	SELF_IMAGE_PRE	0.131	0.821
3	GAD7AFTERNUMBER	GAD7BEFORENUMBER	2.498	0.654
4	PHQ8AFTERNUMBER	PHQ8BEFORENUMBER	4.229	0.493
5	WHO5AFTERNUMBER	WHO5BEFORENUMBER	32.875	0.505
6	SELFESTEEMAFTERNUMBER	SELFESTEEMBEFORENUMBER	3.625	0.834
7	SELFEFFICACYAFTERNUMBER	SELFEFFICACYBEFORENUMBER	7.612	0.765

**Table 5:** Model summary for the 7 simple linear regression analyses.

Number of Regression Analysis	R	R Square	Adjusted R-Squared	F Change	df1	df2	Sig. F Change
1	0.721	0.52	0.514	269.899	1	249	<0.001
2	0.9	0.81	0.804	1059.223	1	249	<0.001
3	0.679	0.462	0.459	213.453	1	249	<0.001
4	0.549	0.301	0.299	107.434	1	249	<0.001
5	0.591	0.349	0.347	133.759	1	249	<0.001
6	0.867	0.752	0.751	756.901	1	249	<0.001
7	0.833	0.694	0.693	564.797	1	249	<0.001

## Data Availability

Not applicable.

## Abbreviations

Abbreviation	Definition of the abbreviations
SCS	Second Chance School
GAD7	The value extracted from the questionnaire GAD-7 (the variable includes observations, regardless of whether they are before or after the studies)
PHQ8	The value extracted from the questionnaire PHQ-8 (the variable includes observations, regardless of whether they are before or after the studies)
WHO5	The value extracted from the questionnaire WHO-5 (the variable includes observations, regardless of whether they are before or after the studies)
SELFEFF	The value extracted from the questionnaire Generalized Self-Efficacy Scale (the variable includes observations, regardless of whether they are before or after the studies)
SELFEST	The value extracted from the questionnaire Rosenberg Self-Esteem Scale (RSES) (the variable includes observations, regardless of whether they are before or after the studies)
MENTAL_HEALTH_POST	The value (factor scores) of mental health after the completion of the studies
MENTAL_HEALTH_PRE	The value (factor scores) of mental health at the beginning of the studies
SELF_IMAGE_POST	The value (factor scores) of self-image after the completion of the studies
SELF_IMAGE_PRE	The value (factor scores) of self-image at the beginning of the studies
GAD7AFTERNUMBER	The value extracted from the questionnaire GAD-7 after the completion of the studies
GAD7BEFORENUMBER	The value extracted from the questionnaire GAD-7 at the beginning of the studies
PHQ8AFTERNUMBER	The value extracted from the questionnaire PHQ-8 after the completion of the studies
PHQ8BEFORENUMBER	The value extracted from the questionnaire PHQ-8 at the beginning of the studies
WHO5AFTERNUMBER	The value extracted from the questionnaire WHO-5 after the completion of the studies
WHO5BEFORENUMBER	The value extracted from the questionnaire WHO-5 at the beginning of the studies
SELFESTEEMAFTERNUMBER	The value extracted from the questionnaire Rosenberg Self-Esteem Scale (RSES) after the completion of the studies
SELFESTEEMBEFORENUMBER	The value extracted from the questionnaire Rosenberg Self-Esteem Scale (RSES) at the beginning of the studies
SELFEFFICACYAFTERNUMBER	The value extracted from the questionnaire Generalized Self-Efficacy Scale after the completion of the studies
SELFEFFICACYBEFORENUMBER	The value extracted from the questionnaire Generalized Self-Efficacy Scale at the beginning of the studies
